# Detailed Visual Memory Capacity Is Present Early in Childhood

**DOI:** 10.1162/OPMI_a_00014

**Published:** 2017-12-01

**Authors:** Katrina Ferrara, Sarah Furlong, Soojin Park, Barbara Landau

**Affiliations:** 1 Department of Cognitive Science, Johns Hopkins University; 2 Department of Psychology, Yonsei University

**Keywords:** visual memory, cognitive development, memory capacity, object recognition, memory fidelity

## Abstract

Previous studies have shown that adults are able to remember more than 1,000 images with great detail. However, little is known about the development of this visual capacity, nor its presence early in life. This study tests the level of detail of young children’s memory for a large number of items, adapting the method of Brady, Konkle, Alvarez, and Oliva (2008). Four- and six-year-old children were shown more than 100 images of everyday objects. They were then tested for recognition of familiar items in a binary decision task. The identity of the foil test item was manipulated in three conditions (Category, Exemplar, and State). Children demonstrated high accuracy across all conditions, remembering not only the basic-level category (Category), but also unique details (Exemplar), and information about position and arrangement of parts (State). These findings demonstrate that children spontaneously encode a high degree of visual detail. Early in life, visual memory exhibits high fidelity and extends over a large set of items.

## INTRODUCTION

The capacity to form and retrieve visual representations of objects is a central aspect of human cognition. Preserving detailed aspects of these object representations holds functional importance for everyday activities: We may want to remember which kitchen implement had been washed (a spoon or a spatula?), which particular category member was used in a recipe (serrated knife or a smooth blade?), or in what particular state a given individual item was left (toaster oven open or closed?). In support of these commonplace tasks, studies have shown that the information capacity of visual memory in adults is surprisingly detailed as well as impressively large (Brady, Konkle, Alvarez, & Oliva, 2008; Konkle, Brady, Alvarez, & Oliva, 2010).

Is this capacity the outcome of adults’ massive exposure to objects and their regular engagement in tasks that require object discrimination based on subtle differences? Or does it reflect a signature characteristic of the human visual memory system, present even without lifelong practice? If so, this capacity should be present in children, despite their more limited need to remember detailed differences among objects. Little work has been done to investigate the overall capacity and level of detail inherent in children’s visual memory, and whether the capacity itself undergoes developmental change. These questions are explored in the present research by testing the extent of children’s visual memory for images of objects.

Existing research shows that adults have an impressive ability to remember previously viewed images. A seminal study in the 1970s found that after viewing 10,000 scenes for only 5 seconds each, participants were 83% correct in identifying which of two images they had seen (Standing, 1973). Further work has shown that, even after several hours, adults can remember details of particular objects they saw, suggesting that their memory stores more than simply the gist of the image (Wolfe, 1998). For example, adults are able to distinguish between a target object and similar distractors that belong to the same basic-level category (Castelhano & Henderson, 2005; Hollingworth, 2004). More recently, Brady et al. (2008) exposed adults to a large number of objects, and then tested their memory using a forced-choice task in which the identity of the foil was carefully manipulated. Participants first viewed images of 2,500 objects. At test, they were shown two images, only one of which they had seen before. The target image was paired with either an object from a different category (e.g., a sofa vs. a banana), an object from the same basic-level category (e.g., a blue train vs. a green train), or the same object in a different state (e.g., an upright bucket vs. the same bucket on its side). Across all three conditions, performance was quite high (92%, 88%, and 87%, respectively). These findings show that adults can store thousands of object representations (Brady et al., 2008; Konkle et al., 2010).

In contrast to the breadth of research in the adult literature, few studies have probed the nature of visual memory capacity in childhood. Some have used visual recognition tests to assess children’s memory (e.g., Luciana & Nelson, 1998; Rose, Feldman, Futterweit, & Jankowski, 1997), but these studies have typically focused on the ability to identify a familiar target among a small set of alternatives (e.g., four items). Standardized measures include assessments of visual memory, but these are not often normed below 5 years of age (e.g., the Benton Visual Retention Test; Sivan, 1992), or only focus on specific categories (e.g., faces or shapes; Test of Memory and Learning; Reynolds & Bigler, 1994; Children’s Memory Scale; Cohen, 1997). Another widely used tool focuses on recall of a single complex figure (i.e., the Rey-Osterrieth Complex Figure; Osterrieth, 1944; Rey, 1941), and performance is measured by having children copy the figure by drawing (e.g., Akshoomoff & Stiles, 1995). This may underestimate the amount of visual detail that can be remembered, especially in children younger than 6 years (Frisk, Jakobson, Knight, & Robertson, 2005).

Could young children’s visual memories be as fine-grained as those of adults? Or do children begin with coarser visual memory representations, which then develop in level of detail? Visual recognition memory in infants has been studied extensively (for review, see Rose, Feldman, & Jankowski, 2004). In terms of short-term iconic visual memory, 6-month-olds demonstrate a 5-object memory capacity, which nearly matches adults’ 6-object capacity (Blaser & Kaldy, 2010). Additional evidence suggests that infants retain at least some degree of perceptual detail in object representations over longer delays. At 5–6 months of age, infants are able to recognize, after a delay of two days, which of two abstract patterns they had seen (Fagan, 1973). Three-month-olds demonstrate recognition of faces that they have been habituated to after a delay of 24 hours (Pascalis, de Haan, Nelson, & de Schonen, 1998). Research using the deferred imitation paradigm, in which participants reproduce actions using props (e.g., Meltzoff, 1985), has shown that infants as young as 14 months are capable of remembering actions based on a single observation experience (Bauer, Larkina, & Deocampo, 2010). In work on episodic memory (Tulving, 1983), 2- to 4-year-olds exhibit impressive abilities to remember past events, even after a delay of 24 hours (Simcock & Hayne, 2003). Children as young as 15 months demonstrate episodic-like memory for past events (Newcombe, Balcomb, Ferrara, Hansen, & Koski, 2014), and this ability further improves during middle childhood (Ghetti, Mirandola, Angelini, Corndoli, & Ciaramelli, 2011). Collectively these studies suggest the possibility that visual memory capacity could be well-developed early in life. However, much of this work has focused on infants and to our knowledge no study has yet attempted to measure the sheer capacity of visual memory in childhood.

In the present work, we draw upon the experiment of Brady et al. (2008), which leverages subtle differences in stimuli to provide insights about the capacity of adult visual memory. The current work tests 4- and 6-year-olds’ memory for a large set of images (116). We aim to gain a better understanding of children’s memory capacity by pushing both the number of items to be remembered (quantity), as well as the specificity of information remembered per item (fidelity) (Konkle et al., 2010). As numerous studies have shown that adults have a massive capacity for visual information, it is possible that this is a fundamental ability present early in life. If so, we predict that visual memory in children will show the signature characteristics of the mature system (i.e., detailed memory for a large set of items).

## METHOD

### Participants

Forty-eight children participated in this study. They were divided into two groups: 4-year-olds (*n* = 24, mean age = 4;6, age range = 4;0–4;11, *SD* = 0;3, 12 females) and 6-year-olds (*n* = 24, mean age = 6;6, age range = 6;0–6;11, *SD* = 0;4, 12 females). Two additional children were tested but were not included in the subsequent analyses due to non participation (*n* = 1) or equipment failure (*n* = 1). The study was approved by the local Institutional Review Board. Children provided verbal assent for their participation and caregivers provided written informed consent. All participants had parent-reported normal or corrected-to-normal vision. Children were recruited via local parent groups and preschools. At the end of the study, children chose a small toy to take home.

### Stimuli and Design

**Figure 1.**
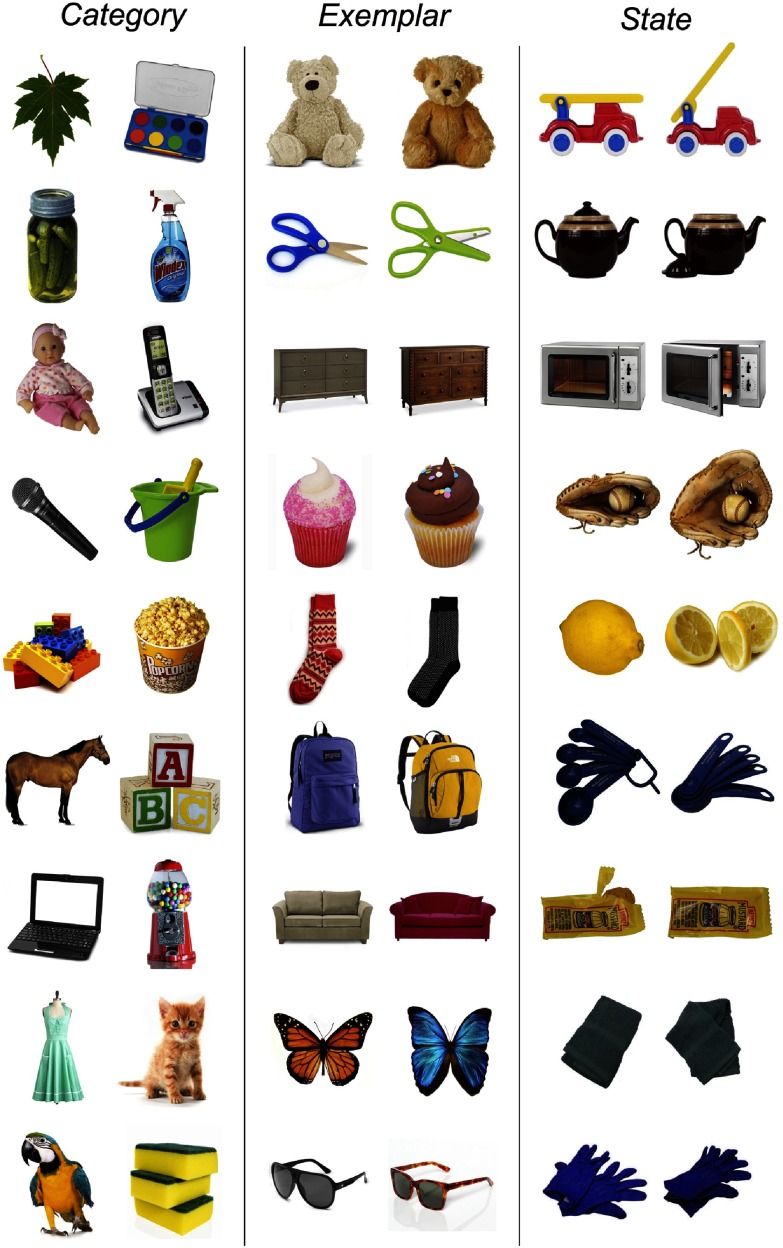
**Nine example test pairs presented during the two-alternative forced-choice task for the three conditions (Category, Exemplar, and State).** There were 50 test pairs per condition. The complete stimulus set is available at http://parklab.johnshopkins.edu/IMAGE_SETS_2.html.

Stimuli were drawn from the image stimulus set of Brady et al. (2008), which includes objects from distinct basic-level categories in order to minimize conceptual interference (Konkle et al., 2010; Koutstaal & Schacter, 1997). In some cases particular items were revised in order to be recognizable to young children (e.g., a cupcake instead of a contact lens case). These revised stimuli were gathered via Internet searches using Google Image Search. The complete set of stimuli is available on our website (Ferrara, Furlong, Park, & Landau, 2017a). Sample stimuli are shown in Figure 1.

Within each age group, eight children were assigned to one of three conditions (Category, Exemplar, or State). These conditions were modeled on those of Brady et al. (2008) and systematically varied the level of detail required for accurate performance at test. In the Category condition, a previously seen item was paired with a new item that was from a different basic-level category (e.g., a baby doll vs. a phone). In this condition, remembering the basic-level category of the previously seen item is sufficient to choose the correct image. In the Exemplar condition, a previously seen item was paired with a new item from the same basic-level category (e.g., a purple backpack vs. a yellow backpack). Accuracy in this condition would require remembering the particular item that had been seen; remembering only the basic-level category of the previously seen item would result in chance performance at test. Lastly, in the State condition, the previously seen item was paired with a new item that portrayed the same object but in a different pose or state (e.g., a whole lemon vs. a sliced lemon). In this condition, memory for just the category or even the specific identity of the object would be inadequate to make the correct choice.

### Procedure

Children were seated at a laptop computer at a comfortable viewing distance. The study in volved two parts: familiarization (on average, 15.35 minutes) and test (on average, 20.10 min utes).^1^ During familiarization, children viewed a continuous stream of 116 images on a computer screen. Images were shown for 3 seconds each. In order to maintain children’s attention, a one-back repetition detection task was used (called “catch the sneaky picture”). Children were instructed to monitor the stream of images for “sneaky” (repeated) images that “took two turns in a row.” They indicated that they saw a “sneaky one” by clapping. Whenever they identified a repeated picture they earned a sticker. Children achieved 100% accuracy in detecting the repeated image. Eight items in the stream were designated as repeats, and none of the repeated items were used at test. Repeat images were inserted into the stream randomly, with the constraint that at least three nonrepeated images must intervene. The remaining 100 nonrepeated images were presented in a randomized order for each participant.

Children were not instructed to remember the objects they would see. Rather, they were simply told to “watch for the sneaky picture.” This aspect of the procedure differs from that of Brady et al. (2008) and Konkle et al. (2010), in which adult participants were explicitly informed at the start of the study that they should try to remember all the items they saw. We opted not to explicitly instruct participants in this way, due to concerns about variable use of metacognitive strategies by children of this age (Bjorklund, Dukes, & Brown, 2009).

At test, participants completed 50 trials of a two-alternative forced choice decision. Each item that was viewed during familiarization was paired with a new image, and par ticipants were asked to indicate which one they had seen before. After every 10 completed test trials children earned a sticker. They were permitted to proceed at their own pace. The identity of the foil image was determined by the condition assigned to the particular participant (Category, Exemplar, or State). The test trials were presented in a randomized order for each participant.

As in Brady et al. (2008), the 50 pairs of images used to test each condition remained consistent across participants (e.g., in the Category condition, the leaf was always paired with the paint box, the doll was always paired with the phone, etc.). Within each pair, the image assigned to be familiarized and the image assigned to be the test foil were counterbalanced across participants (e.g., some participants were familiarized to the leaf and saw it paired with the paint box at test, while others were familiarized to the paint box and saw it paired with the leaf at test). The 50 familiarized images that were later tested were dis tributed evenly throughout the entire stream of images during the familiarization portion of the study.

## RESULTS

**Figure 2.**
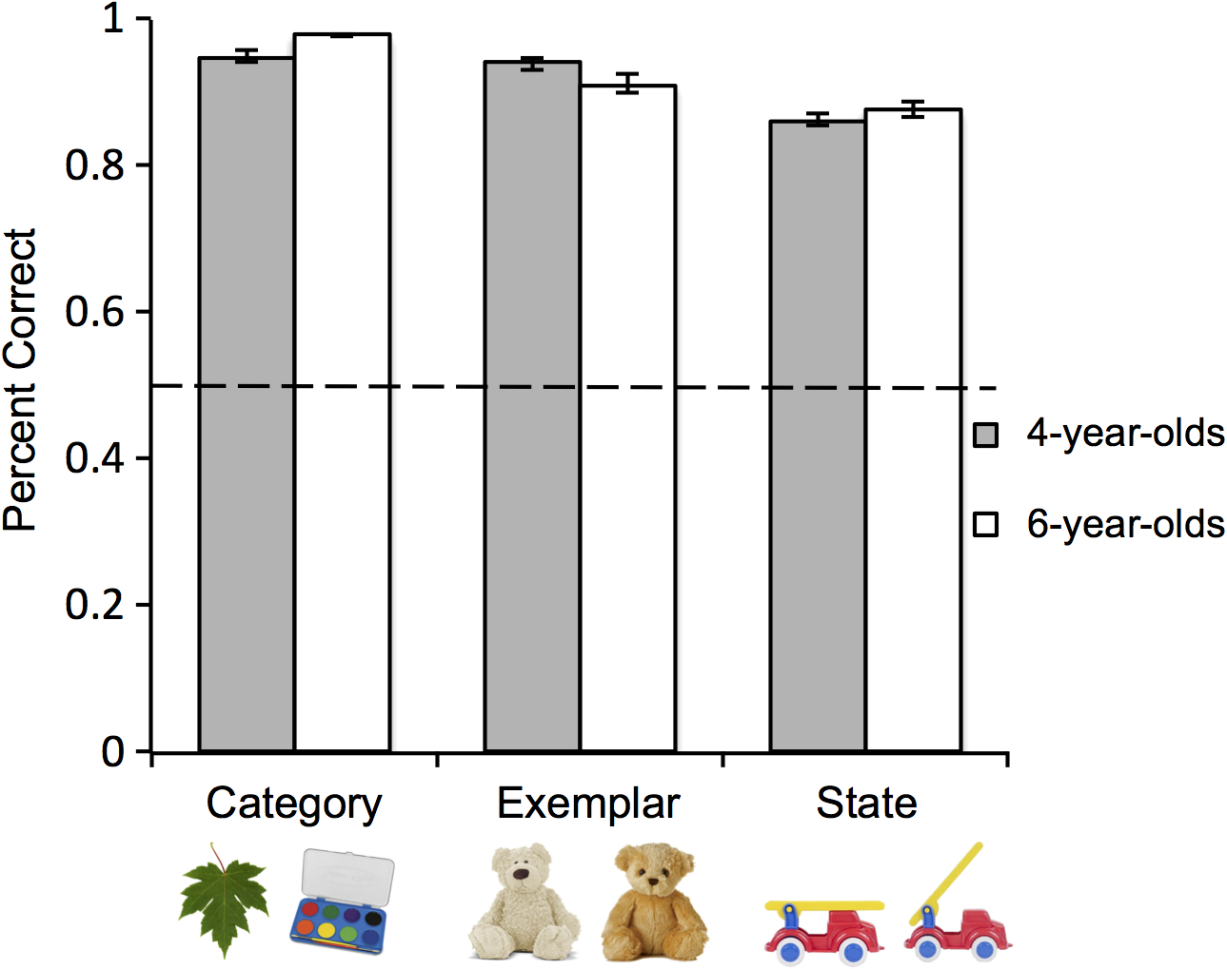
**Percent correct in the Category, Exemplar, and State conditions for 4-year-olds and 6-year-olds.** Error bars reflect one standard error of the mean (*SE*). Chance performance (50%) is indicated by the dashed line.

Children in both age groups were highly accurate on the forced-choice decision at test across all three conditions (Figure 2). In the Category condition, the mean percent correct for 4-year-olds was 95.00% (*SE* = 0.86%) and the mean percent correct for 6-year-olds was 98.00% (*SE* = 0.23%). In the Exemplar condition, the mean percent correct for 4-year-olds was 94.00% (*SE* = 1.00%), and the mean percent correct for 6-year-olds was 91.13% (*SE* = 1.17%). In the State condition, the mean percent correct for 4-year-olds was 86.25% (*SE* = 0.85%) and the mean percent correct for 6-year-olds was 87.75% (*SE* = 0.99%). No gender differences in accuracy were found for either the 4-year-olds, two-tailed *t*(22) = –0.35, *p* = .73, Cohen’s *d* = –0.14, or 6-year-olds, *t*(22) = 0.30, *p* = .77, Cohen’s *d* = 0.12.

Participant accuracy (percent correct) was submitted to a 3 (Condition: Category, Exemplar, State) x 2 (Age: 4-year-olds, 6-year-olds) analysis of variance (ANOVA) with Condition and Age as between-subjects factors. This analysis yielded a main effect of condition, *F*(2, 42) = 7.05, *p* = .002, η_*p*_^2^ = .25, indicating that performance was affected by the level of detail required to remember the familiar item at test. The main effect of Age was nonsignificant, *F*(1, 42) = .08, *p* = .78, η_*p*_^2^ = .002, as was the interaction between Age and Condition, *F*(2, 42) = .69, *p* = .51, η_*p*_^2^ = .03. Thus, performance did not significantly improve with age, and the different conditions did not differentially affect accuracy for 4-year-olds versus 6-year-olds. Analyses using the Scheff post hoc criterion for significance showed that the Category condition did not elicit significantly higher accuracy than the Exemplar condition, *F*(2, 42) = 1.57, *p* = .32, 95% confidence interval, CI = [−.03, .10]. The difference between accuracy in the Exemplar and State conditions was also not significant, *F*(2, 42) = 2.45, *p* = .10, 95% CI = [−.01, .12]. Comparison of the Category and State conditions, however, showed that the Category condition elicited significantly higher accuracy than State, *F*(2, 42) = 6.97, *p* = .002, 95% CI = [−.03, .16]. The greater accuracy in the Category condition compared to State stands as a replication of Brady et al.’s (2008) findings with adults. This indicates that both children and adults were able to store and retrieve fewer items when specific states had to be remembered, compared to simply remembering the object’s category.

One possibility is that the visual similarity of the item pairs in the three conditions varied such that poorer performance in the State condition was due to higher visual similarity between targets and their foils. We used a phase correlation algorithm to quantify the relative visual similarity between two test images. We found that the State condition had the highest overall interitem similarity, but higher image similarity values for specific item pairs did not predict behavioral performance in terms of accuracy (see Supplemental Figure 1 and Supplemental Table 1; Ferrara, Furlong, Park, & Landau, 2017b).

We also considered the potential effect of the number of intervening items (“item delay”) on performance at test. Each participant was exposed to the 100 familiarization images in a randomized order, and the 50 test pairs were also presented in a randomized order. From these orders we computed the number of intervening items between the point at which an item was viewed during familiarization and when it was viewed again at test. If item delay affected accuracy, this would be reflected by a decrease in performance with the increasing number of intervening items. No effects of item delay were found (see Supplemental Figure 2; Ferrara et al., 2017b).

Although the omnibus ANOVA did not reveal a main effect of age, we further explored potential age effects by conducting linear regression analyses to determine whether exact age (measured in terms of years, months, and days) significantly predicted accuracy at test. These analyses showed no significant effects of age for any of the conditions (see Supplemental Figure 3 and Supplemental Table 2; Ferrara et al., 2017b). We also considered the number of test pairs for which children did not obtain 100% accuracy in each condition (see Supplemental Figure 4; Ferrara et al., 2017b). Four- and six-year-olds differed only in the category condition, where 4-year-olds had significantly more test items that fell below 100% accuracy, χ^2^(2, *n* = 100) = 6.45, *p* = .01.

Given that both 4- and 6-year-olds are clearly storing a large amount of visual detail in order to achieve such high levels of performance, we sought to quantify the capacity of children’s visual memory. As noted by Brady et al. (2008), true estimates of memory capacity take into account not only the number of items stored, but also the amount of information that must be remembered per item. To quantify memory capacity, Brady et al. (2008) analyzed their adult data using a model by Landauer (1986). This model is not based on visual similarity, and instead assigns each picture a random code (*b* bits long) and calculates the number of bits required to correctly make a decision about which image has been seen and which has not. Errors occur when two images are mistakenly assigned the same code. The model provides an estimate of the optimal code length (bits per item) based on the total number of items in the image set and the percentage correct on a two-alternative forced-choice memory test.^2^ Adults in Brady et al. (2008) obtained 93% in the Novel condition (in our experiment called the Category condition). Using Landauer’s model, Brady et al. found that the optimal code for this performance would require 13.8 bits per item. We applied Landauer’s model to our own data to see if a comparable result would be obtained, despite the fact that the image set of the present study is much smaller and children could well have a capacity for far fewer bits than adults. For our Category condition, with an image set of 100 items and 96.5% correct (for all children tested), the optimal code for this performance would require 10.43 bits per item. This is close to the number of bits per item estimated by Brady et al. for their adult participants. As Brady et al. noted, their own empirically derived value is comparable with estimates of 10–14 bits needed to model adult performance in previous large-scale experiments with adults (Landauer, 1986). It is even more striking that the estimated bits per item for our child data falls within this range.

We also modeled the Exemplar and State data following the assumptions laid out by Brady et al. (2008), which assume a hierarchical organization of memory, where the category bits appear first and additional bits of information then specify the exemplar or state of the item in that category. The exemplar bits are assumed to be nonoverlapping with the state bits in the hierarchy. For example, as Brady et al. (2008) assumed, two additional bits are required to code a particular exemplar (12.43 bits per item, in comparison to 10.43 bits per item to code a particular category) and two more are required to code a particular state (14.43 bits per item). Given that the State condition will require the highest number of bits per item, we can use that number as an estimate of the representational capacity of children’s visual memory, assuming that all items are coded by the optimal set of features. That estimate is a massive number ≈ 22,000 (2^14.43^).^3^ This analysis does not tell us the true visual information capacity of the system (Brady et al., 2008), but serves as a formal demonstration of the impressively large memory capacity that must exist to encode the many unique exemplar and state features and obtain the high rates of accuracy observed for both children and adults. Overall, these results point to the remarkable ability of 4- and 6-year-olds to represent fine-grained details of previously seen items, encompassing variation across objects in a single basic-level category, and variation within an individual object in that category.

## DISCUSSION

The present study demonstrates impressive visual memory performance by children as young as 4 years of age, both in terms of the large number of items and the level of detail required for recognition. Children showed accurate recognition not only for the basic-level category of the item they saw (Category), but also for unique featural details (Exemplar), and specific information about position and arrangement of parts (State). As was found previously with adults, performance declined slightly in the State condition (Brady et al., 2008). Memory performance between 4- and 6-year-olds was largely similar. Whether the same levels of performance (and hence estimated visual memory capacity) holds for children younger or older than this age range awaits further study. Overall, these findings demonstrate that the fidelity of children’s visual memory shows the signature characteristics of the mature system—detailed and highly accurate visual memory that extends over a large set of items.

This work highlights clear developmental precursors to the massive visual memory capacity that has been shown in studies of adults. To be sure, those studies included more than 1,000 images while the present includes only 116. Even so, the abbreviated constraints of the current study show that children retain details for a large number of items, seen only for 3 seconds each. The estimated number of bits per item based on Landauer’s model (Landauer, 1986) was comparable to that which Brady et al. (2008) found for adults. This finding is surprising, given the large developmental differences in other aspects of memory (e.g., working memory [Gathercole, 1999; Hale, Bronik, & Fry, 1997; Luciana & Nelson, 1998] and episodic memory [Brainerd, Holliday, & Reyna, 2004; DeMaster & Ghetti, 2013; Lloyd, Doydum, & Newcombe, 2009]). Notably, high levels of accuracy were observed even when children were not instructed to remember anything about the images they saw—during familiarization they were fully engaged in the “catch the sneaky picture” game. This indicates that explicit strategies are unlikely to account for performance. These data also suggest that the retrieval and comparison processes necessary to successfully perform the two-alternative forced-choice test are relatively well-developed in young children. Furthermore, it is unlikely that children were able to linguistically encode nuanced differences among the stimuli within the brief duration for which each image was displayed. Collectively, these findings indicate that the storage of fine-grained visual information is a fairly automatic and rapid process in early childhood.

In our study, children showed exceptional memory capacity for even the subtle visual differences presented in the State condition. This observation is consistent with findings from infant studies, which indicate that object heterogeneity can affect memory performance. When habituated to two objects that differ in color, pattern, and texture, 7-month-olds respond to a change in the number of objects present. But when habituated to two identical objects, infants fail to respond to a change in object number (Feigenson & Carey, 2005). This suggests that in infancy, the distinctiveness of visual detail is an important element toward forming separate representations for multiple objects, which holds repercussions for further cognitive processing, such as representation of numerosity. Additional work has shown that when the perceptual contrast between objects is highly salient, infants show improved working memory capacity (Zosh & Feigenson, 2015). The current findings expand upon this developmental account; here we observe high levels of accuracy, even when interitem similarity is quite high. Building upon the attention to visual detail that they demonstrate as infants, 4-year-olds can form distinct and enduring object representations based on slight variations in visual appearance.

It is important to note that although object memory is highly detailed and accurate in children and adults, this does not hold for other types of visual stimuli, such as images of scenes. On scene memory tasks, both children and adults make consistent errors that are not faithful to the original stimulus. Research has shown that children as young as 4 years of age demonstrate the phenomenon of boundary extension (false memory beyond the edges of a view of a scene; Kreindel & Intraub, 2016), which has also been shown in adults (Intraub, 2010; Intraub & Richardson, 1989). Thus, the highly detailed nature of children’s visual memory reported here supports aspects of object recognition, but may not extend to other cognitive functions, such as scene representation.

These findings suggest several avenues of further investigation. First, what is the visual memory capacity of infants? Could it be within the same range shown by the 4- and 6-year-olds in our study? It may be possible to answer this question by using measures such as looking time to determine whether items from a relatively large set of objects are later recognized as different from foils that differ in terms of category, exemplar, and/or state. Second, do the effects we’ve observed persist over longer periods of time? To find out, one could test children’s ability to recognize foils over delays of hours, or even days. Capacity could be further investigated by increasing the set size above 100 images. The present data suggest that children might obtain high levels of accuracy on even larger sets of images, as analyses of item delay did not reveal any decrease in accuracy as the number of intervening items increased.

Lastly, this study raises questions about the neural basis of the ability to form and maintain visual object representations. This work could be expanded to tasks that include neuroimaging measures, which could further our understanding of the maturation and functioning of key regions that underlie human memory, such as the dentate gyrus and hippocampus for pattern separation and completion (Bakker, Kirwan, Miller, & Stark, 2008; Hunsaker & Kesner, 2013; Jabès & Nelson, 2015). In sum, our findings demonstrate that children possess impressive visual memory capacity even early in life, and provide intriguing avenues for future research.

## ACKNOWLEDGMENTS

We are very grateful to all the families who took part in this study. We thank Matthew Levine for his technical assistance in developing the experimental MATLAB scripts.

This research was supported by an Integrative Graduate Education and Research Traineeship through the National Science Foundation (DGE 0549379 to K. Ferrara), a T32 Postdoctoral Research Fellowship through the National Institutes of Health (5T32 HD 046388 to K. Ferrara), a Johns Hopkins University Provost Undergraduate Research Award (to S. Furlong), a grant from the National Institutes of Health (NINDS RO1 050876 to B. Landau), and a grant from the National Institutes of Health (NIH R01 EY026042 to S. Park).

## AUTHOR CONTRIBUTIONS

SP and BL contributed equally to the research design, data analysis, and writing of the paper. All authors contributed to the study concept and design. Testing and data collection were performed by KF and SF. KF and SF performed data analysis and interpretation under the super vision of BL and SP. KF and SF drafted the manuscript, and BL and SP provided critical revisions. All authors approved the final version of the manuscript for submission.

## Notes

^1^ Durations are given as averages because children were permitted to proceed at their own pace. No individual participant exceeded +/– 4 minutes of the given familiarization and test durations.^2^ The number of bits is related to memory performance by the following equation: *b* = –log_2_ [1 – (2*p* –1)^1/*n*^], where *p* is the percentage correct and *n* is the number of items in memory.^3^ Two objects (target and foil), with 14.43 bits/object.
