# Supplementary material for: Detailed Visual Memory Capacity Is Present Early in Childhood
Source: Open Mind (Camb). 2017 Dec 1;1(3):136–47. doi: 10.1162/OPMI_a_00014 (PMC6436559; doi:10.1162/OPMI_a_00014)
Supplement: Supplementary file 1 [file opmi-03-136-s001.pdf]

Ferrara, K., Furlong, S., Park, S., & Landau, B. (2017). Detailed Visual Memory Capacity Is Present Early in Childhood. *Open Mind: Discoveries in Cognitive Science*, *1*(3), 136–147. https://doi.org/10.1162/opmi_a_00014

Supplementary Material

One possibility is that the visual similarity of the item pairs in the three conditions varied such that poorer performance in the State condition was due to higher visual similarity between targets and their foils. To evaluate this possibility, we used a phase correlation template-matching algorithm to quantify the relative visual similarity between the two images of each test pair across the three conditions. This was based on normalized cross correlation of the data in the Fourier domain, commonly used for image registration in computer vision (Szeliski, 2006). A similarity value was obtained for each image pair, where higher values correspond to higher inter-item similarity. Independent samples t-tests confirmed that the stimuli in the Exemplar condition (M = .048, SE = .003) had higher similarity values than those in the Category condition (M = .037, SE = .002), two-tailed $t(49) = 2.94$, $p = .005$, Cohen's $d = 0.60$, and that the stimuli in the State condition (M = .073, SE = .011) had higher values than those in the Exemplar condition, two-tailed $t(49) = 2.15$, $p = .036$, Cohen's $d = 0.43$ (Supplementary Figure 1). These data indicate that the image test pairs in the Category condition were the least visually similar to one another, those in the State condition were the most visually similar, and the Exemplar image pairs were of intermediate visual similarity. This is consistent with the interpretation that the conditions increase in difficulty as visual similarity among objects increases, although the difference in performance between the Category and Exemplar conditions did not reach significance at the group level.

[Insert Supplementary Figure 1 here]

Supplementary Figure 1. Average image similarity values for the test pairs in each of the experimental conditions (Category, Exemplar, and State). Significance indicated by *t*-test, two-tailed, $p < .05$.

To determine whether differences in visual similarity values predicted participants' accuracy at test for individual image pairs (e.g., whole lemon vs. sliced lemon), we carried out linear regression analyses. We predicted that higher image similarity values would lead to lower accuracy, reflected by negative correlations. None of these analyses reached significance for either 4-year-olds or 6-year-olds (Supplementary Table 1). Thus, although there are overall group trends showing that accuracy decreases from the Category to Exemplar to State conditions and that image similarity increases across these conditions, degree of visual similarity does not predict performance on individual items.

Supplementary Table 1. Linear regression using image pair visual similarity values as the independent variable and accuracy as the dependent variable. The table displays standardized regression coefficients (β), 95% confidence intervals (CI) for β (in brackets), *t* values, and *p* values (two-tailed).

	4-year-olds			6-year-olds		
	β [95% CI]	*t*	*p*	β [95% CI]	*t*	*p*
Category	–0.22 [–2.82, .35]	–1.56	.124	–0.12 [–1.24, .52]	–0.82	.416
Exemplar	–0.05 [–.75, 1.06]	–0.35	.730	–0.28 [–2.83, –.01]	–2.01	.060
State	–0.03 [–.18, .15]	–0.18	.862	–0.10 [–.23, .11]	–.70	.485

We also reviewed performance for individual pairs of images and noted that performance dropped below the chance level of 50% for two item pairs in particular. Accuracy for the gloves pair (shown in Figure 1, State condition, bottom row) was 37.50% for 4 year-olds (75% for 6-year-olds); accuracy for the towels pair (Figure 1, State condition, second from the bottom) was 25% for 6 year-olds (70% for 4-year-olds). These were the only two cases across all item pairs and conditions for which performance was below chance (and even this was not consistent across the age groups). In the Category and Exemplar conditions, performance for all of the individual item pairs was above chance for both age groups. We noted that both the glove and towel State pairs appear to have a high degree of visual similarity between familiarized item and foil, which could have made them more difficult for participants. To investigate this further, we ranked all 150 test pairs, regardless of their condition, by their image similarity value (described above), from highest to lowest. If the gloves and towels were most difficult because they had particularly high image similarity, we predicted that they would be among the top test pairs on this list. We found that the gloves pair had a similarity value of 0.053, and was #42 out of 150 on the ranked list. The towels pair had a similarity value of 0.034, and was #101 on the list. The average similarity value of all the test pairs was 0.049 (SE = 0.004). Thus, these two test pairs do not particularly stand out in terms of high image similarity as we predicted; the gloves pair is just above average and the towels pair is below average. It appears that poor performance on these pairs is likely due to random variation, which is further supported by the observation that the below chance performance for the two pairs was not consistent across the two age groups.

We considered the effect of the number of intervening items on performance at test (Supplementary Figure 2). We computed the number of intervening items between the point at which an item was viewed during familiarization and when it was viewed again at test. If this item delay affected accuracy, this would be reflected by a decrease in performance as the number of intervening items increases.

[Insert Supplementary Figure 2 here]

Supplementary Figure 2. Accuracy at test (percent correct), broken down by the number of intervening items between the point at which the item was viewed during familiarization and when it was viewed again at test, for the A) Category, B) Exemplar, and C) State conditions, shown separately for 4-year-olds and 6-year-olds. Error bars reflect one standard error of the mean (*SE*). No differences were found between the binned groups of number of items back (e.g., between 1-25 items and 26-50 items) or between 4-year-olds and 6-year-olds (all ts < 1, n.s.).

To evaluate the relationship between performance and age (Supplementary Figure 3, Table 2), linear regression analyses were used to determine whether age significantly predicted accuracy at test. Exact age was measured in terms of years, months, and days (converted to a decimal value). These analyses showed no significant effects of age for any of the conditions.

[Insert Supplementary Figure 3 here]

Supplementary Figure 3. A) Plot of accuracy at test (percent correct, *y* axis) in the Category condition against exact age (*x* axis). B) Plot of accuracy at test in the Exemplar

condition against exact age. C) Plot of accuracy at test in the State condition against exact age.

Supplementary Table 2. Linear regression results using exact age as the independent variable and accuracy as the dependent variable. The table displays standardized regression coefficients (β), 95% confidence intervals (CI) for β (in brackets), *t* values, and *p* values (two-tailed).

	β [95% CI]	*t*	*p*
Category	0.31 [–.01, .05]	1.23	0.237
Exemplar	–0.16 [–.05, .03]	–0.60	0.561
State	0.19 [–0.19, .04]	0.74	0.473

We also considered individual accuracy for those test items in each condition for which children did not obtain 100% accuracy (Supplementary Figure 4). In the Category condition, 4-year-olds had significantly more test items that were below perfect performance than 6-year-olds, χ^2 (2, n = 100) = 6.45, *p* = .01. The age groups did not differ in the Exemplar condition, χ^2 (2, n = 100) = 1.01, *p* = .31, or the State condition, χ^2 (2, n = 100) = 1.05, *p* = .31. It is unclear why this difference emerged in the Category condition in particular; this may be due to random group variation or increased memory capacity in 6-year-olds for multiple object categories. Linear regression analyses revealed that exact age did not significantly predict performance on items below 100% accuracy for any of the conditions (Supplementary Table 3).

[Insert Supplementary Figure 4 here]

Supplementary Figure 4. Number of test items, by condition, for which 4-year-olds and 6-year-olds were below 100% correct. Significance determined by χ^2 test, $p < .05$.

Supplementary Table 3. Linear regression results using exact age as the independent variable and accuracy for items showing less than 100% group performance as the dependent variable. The table displays standardized regression coefficients (β), 95% confidence intervals (CI) for β (in brackets), *t* values, and *p* values (two-tailed).

	β [95% CI]	*t*	*p*
Category	0.05 [–.08, .10]	0.17	-0.868
Exemplar	–0.01 [–.08, .08]	–0.05	0.962
State	0.02 [–.05, .05]	0.08	0.935
